# Migraine and cluster headache show impaired neurosteroids patterns

**DOI:** 10.1186/s10194-019-1005-0

**Published:** 2019-05-27

**Authors:** Angela Koverech, Claudia Cicione, Luana Lionetto, Marta Maestri, Francesco Passariello, Elisabetta Sabbatini, Matilde Capi, Cristiano Maria De Marco, Martina Guglielmetti, Andrea Negro, Luisa Di Menna, Maurizio Simmaco, Ferdinando Nicoletti, Paolo Martelletti

**Affiliations:** 1 Department of Clinical and Molecular Medicine, Sapienza University and Regional Referral Headache Centre, Sant’Andrea Hospital, via di Grottarossa 1035-1039, 00189 Rome, Italy; 2 Residency Program of Internal Medicine, School of Medicine and Psychology, Sapienza University, 00189 Rome, Italy; 3 Laboratory of Advanced Molecular Diagnostics, IRCSS Istituto Dermopatico dell’Immacolata, 00167 Rome, Italy; 4 Laboratory of Experimental Immunology, IRCSS Istituto Dermopatico dell’Immacolata, 00167 Rome, Italy; 5 Regional Referral Headache Centre, Sant’Andrea Hospital, 00189 Rome, Italy; 6 Department of Medical, Surgical and Experimental Sciences, University of Sassari, Sassari, Italy; 7 IRCCS Neuromed, 86077 Pozzilli (IS), Italy; 8 Department of Neurosciences, Mental Health and Sensory Organs, Sapienza University, 00189 Rome, Italy; 9 Advanced Molecular Diagnostics Unit, Sant’Andrea Hospital, 00189 Rome, Italy; 10 Department of Physiology and Pharmacology “Vittorio Erspamer”, Sapienza University, 00189 Rome, Italy

**Keywords:** Episodic migraine, Chronic migraine, Cluster headache, Allopregnanolone, Dehydroepiandrosterone

## Abstract

**Background:**

Perturbation of neuronal excitability contributes to migraine. Neurosteroids modulate the activity of γ-aminobutyric acid A and N-methyl-d-aspartate receptors, and might be involved in the pathogenesis of migraine. Here, we measured plasma levels of four neurosteroids, i.e., allopregnanolone, epiallopregnanolone, dehydroepiandrosterone and deydroepiandrosterone sulfate, in patients affected by episodic migraine, chronic migraine, or cluster headache.

**Methods:**

Nineteen female patients affected by episodic migraine, 51 female patients affected by chronic migraine, and 18 male patients affected by cluster headache were recruited to the study. Sex- and age-matched healthy control subjects (31 females and 16 males) were also recruited. Patients were clinically characterized by using validated questionnaires. Plasma neurosteroid levels were measured by liquid chromatography-tandem mass spectrometry.

**Results:**

We found disease-specific changes in neurosteroid levels in our study groups. For example, allopregnanolone levels were significantly increased in episodic migraine and chronic migraine patients than in control subjects, whereas they were reduced in patients affected by cluster headache. Dehydroepiandrosterone and dehydroepiandrosterone sulfate levels were reduced in patients affected by chronic migraine, but did not change in patients affected by cluster headache.

**Conclusion:**

We have shown for the first time that large and disease-specific changes in circulating neurosteroid levels are associated with chronic headache disorders, raising the interesting possibility that fluctuations of neurosteroids at their site of action might shape the natural course of migraine and cluster headache. Whether the observed changes in neurosteroids are genetically determined or rather result from exposure to environmental or intrinsic stressors is unknown. This might also be matter for further investigation because stress is a known triggering factor for headache attacks in both migraineurs and cluster headache patients.

## Introduction

Migraine is a chronic disease with relevant clinical and social implications. The recently released results of the Global Burden of Disease initiative reveal that migraine is the fifth leading chronic disease on the planet, being more prevalent than diabetes [1, 2]. Also, migraine negatively influences quality of life, and is the fourth leading disease per number of years lived with disability [1, 2]. These figures highlight the existing gaps in the preventive and symptomatic treatment of migraine.

Among other predisposing factors, the pathogenesis of migraine and cluster headache appears to be related to an impaired control of neuronal excitability [3]. Hence, unraveling the trans-synaptic and/or intracellular mechanisms that alter neuronal firing in migraine and cluster headache may provide new insights into the pathophysiology of these disorders and pave the way to novel therapeutic interventions. Neurosteroids are endogenous steroids synthesized in the central nervous system (CNS) that modulate neuronal excitability by interacting with either γ-aminobutyric acid A or N-methyl-d-aspartate receptors [4–6]. Neurosteroids are synthesized in glial cells from cholesterol or other precursors via progressive reduction of the A-ring of the steroid molecule [4]. Most of the current research on neurosteroids focuses on allopregnanolone (AP), epiallopregnanolone (EAP), dehydroepiandrosterone (DHEA), and DHEA sulfate (DHEAS). AP-like neurosteroids are potent positive allosteric modulators as well as direct activators of both synaptic and extrasynaptic γ-aminobutyric acid A receptors, and, therefore, they can maximally enhance synaptic phasic inhibition and extrasynaptic tonic inhibition [4]. In contrast, DHEA and DHEAS are weak antagonists of γ-aminobutyric acid A receptor and agonists of N-methyl-d-aspartate receptors [7]. Thus, allopregnanolone and DHEA/DHEAS display opposite effects on neuronal excitability. The clinical relevance of neurosteroids is exemplified by their potential role in the pathophysiology of epilepsy [8, 9]. An imbalance between excitatory and inhibitory neurotransmission leading to a hyperactivation of N-methyl-d-aspartate receptors is considered as a key event in the development of migraneous aura and in mechanisms of nociceptive sensitization underlying the central component of migraneous pain [10, 11]. Owing to their mechanisms of action, neurosteroids might participate to the pathophysiology of migraine particularly under conditions associated with changes in the peripheral production of progesterone and other neurosteroid precursors (e.g., the pre-menstraul period). The association between serum γ-aminobutyric acid levels and the clinical characteristics of migraine suggests a causal link between changes in γ-aminobutyric acid mediated neurotransmission and the pathophysiology of migraine [12]. However, whether neurosteroids are part of this link remains undetermined.

The aim of our study was to measure for the first time circulating levels of neurosteroids in patients with ICHD3-beta confirmed diagnosis [13] of episodic or chronic migraine (EM, CM), medication overuse headache (MOH) and cluster headache (CH), and to explore possible associations with their clinical characteristics.

Migraine and cluster headache are complex and multifaceted clinical diseases, whose conceptual framework has been recently organized by the International Classification of Headache Disorders 3rd Edition beta classification [13]. In particular, migraine is considered as a primary headache and patients are diagnosed as having episodic (EM) or chronic migraine (CM) according to well established clinical parameters [13]. In the International Classification of Headache Disorders 3rd Edition beta classification, medication-overuse headache (MOH) is considered within secondary headaches and thus it is independent from migraine [14]. However, it has been recently proposed that MOH should be more correctly considered a complication of CM, if not even in some cases its natural evolution as CM plus medication overuse (CM + MO) [15, 16]. Cluster headache (CH) is also a primary headache, but it is not classified together with migraine rather as a trigeminal autonomic cephalalgia [13].

## Materials and methods

The protocol has been devised as a cross-sectional study. It complies with the rules set by the Declaration of Helsinki and subsequent amendments, and has been approved by the Azienda Ospedaliero-Universitaria Sant’Andrea Ethics Committee. The study has been carried out between February and December 2017.

### Patients

Patients of both sexes, aging 18–80 years and referring to the Headache Unit at Azienda Ospedaliera-Universitaria S. Andrea, Rome, were recruited for the study. Patients were cognitively able to sign the informed consent, and received the diagnosis of EM, or CM, or CM + MO, or CH according to the International Classification of Headache Disorders 3rd Edition -beta criteria [13]. Patients with cancer, liver failure, renal failure or recent administration of benzodiazepines were excluded from the study. Sex- and age-matched healthy subjects were recruited as controls.

### Study design

After signing the informed consent, patients were enrolled and the following information collected and registered: age, sex, education, comorbidities, actual drug therapy, the presence of a concurrent migraine/headache attack, number of attacks/month, day of the menstrual cycle (women only). Then, validated psychometric and functional questionnaires were provided. In particular, patients were asked to fill the Beck Depression Inventory (BDI), the Migraine Disability Assessment Test (MIDAS; only migraine patients), the Headache Impact test (HIT-6), and the Self-rating Anxiety Scale (SAS). BDI is a validated questionnaire for quantifying the severity of depression. MIDAS provides a precise assessment of migraine-induced disability. HIT-6 measures the impact of headache on daily activities. SAS provides a quantitative assessment of anxiety.

### Biochemistry

After signing informed consent, blood sample was collected, and immediately centrifuged. This occurred in the morning, between 8 AM and 9 AM. Aliquots of plasma were separated and kept at − 20 °C until further analysis.

Circulating levels of AP (ng/mL), EAP (ng/mL), DHEA (ng/mL) and DHEAS (μg/mL) were measured by liquid chromatography-tandem mass spectrometry (LC-MS/MS), using the protocol developed and validated at our institution and previously described [17]. Briefly, the analytes were first derivatized with 2-hydrazinopyridine and extracted from plasma using solid phase extraction. Then, the compounds were separated and detected by LC-MS/MS. A mobile phase of formic acid (0.1%) in water and methanol through a gradient of composition and a flow rate of 0.3/mL/min was used since we demonstrated that results in good separations of the analytes [17]. By using this protocol, linear responses in wide range of concentrations and limits of quantification ranging from 10 (DHEAS) to 40 pg/mL (DHEA) are obtained in < 9 min [17].

### Statistical analysis

Results were statistically analysed by Student’s t test and by one-way ANOVA and Fisher’s LSD as a post-hoc test where appropriate. Possible associations between continuous variables were assessed by the Pearson’s correlation test. Data are presented as means ± S.D.

## Results

**Fig. 1:**
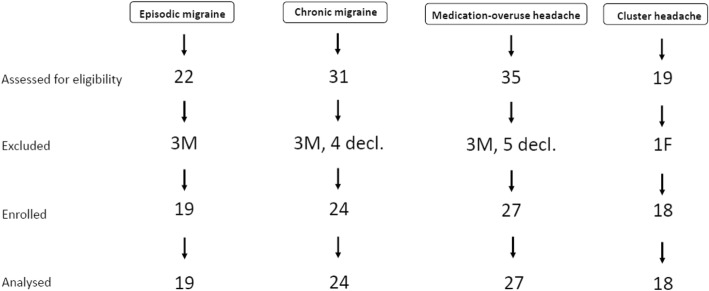
CONSORT flow diagram of the study (M=males; F=female; decl. = declined)

Screening and enrolment procedures are illustrated by the CONSORT flow diagram in Fig. 1.

Considering the imbalanced gender distribution within groups, and the potential role of female sex hormones as precursors of neurosteroids [18], we decided to consider only female patients for EM, CM and MOH, and only male patients for CH. Also, as previously mentioned [15, 16], MOH can be considered as a complication or even as the natural evolution of CM. Therefore, patients with MOH were merged into the CM group.

The control groups included 31 female healthy individuals and 16 male healthy individuals. Patients’ and controls’ characteristics are reported in Table 1

**Table 1:** Demographic and clinical characteristics of the recruited patients affected by episodic migraine, chronic migraine, or cluster headache, and their respective healthy controls

	Episodic migraine	Chronic migraine^a^	Controls (female)	Cluster headache	Controls (male)
n.	19	51	31	18	16
Age (years)	41,6±16,2	51,6±10,9	52,1±17,8	46,2±15,8	58,7±13,8
Menopause	36% (7/19)	62% (32/51)	54% (17/31)	N.A.	N.A.
Sampling during migraine/headache attack	0%	53%	N.A.	39%	N.A.
BDI (n.) *no/mild/mod./severe*	18 *12/4/2*	49 *26/11/7/5*	N.A.	18 *14/3/0/1*	N.A.
SAS (n.) *low/moderate/severe*	17 *14/3/0*	48 *27/18/3*	N.A.	18 *16/2/0*	N.A.
HIT-6 (n.) *mild/mod./relevant/severe*	18 *5/0/0/13*	49 *1/2/2/44*	N.A.	18 *2/5/3/8*	N.A.
MIDAS (n.) *I→IV (low→severe disability)*	18 *2/4/5/7*	49 *4/5/3/37*	N.A.	N.A.	N.A.

^a^including chronic migraine and medication-overuse headache patients

.

### Episodic migraine

Plasma neurosteroid levels fell within the expected range in all our patients and controls (e.g., 3–10 nM corresponding approximately to 1–3.7 ng/ml of AP) [19]. AP and EAP levels were largely increased in patients affected by EM, whereas DHEA levels were reduced by > 40% (Table 2). No changes in DHEAS levels and in the ratio between AP and EAP were found in patients affected by EM with respect to healthy controls (1.9 ± 0.9 vs 1.8 ± 1.2; p = n.s.). Knowing that the ovarian cycle has a strong impact on neurosteroid synthesis [16], we also examined AP, EAP, DHEA, and DHEAS levels in pre- and post-menopausal patients and healthy controls. Differences in AP, EAP, and DHEAS levels between EM patients and controls were maintained when subjects were stratified according to their menopausal status. In addition, no changes in neurosteroid levels were found between pre- and post-menopausal EM patients.

**Table 2:** Plasma neurosteroid levels in patients affected by episodic migraine patients and healthy controls

	Episodic migraine	Controls	*P*
AP (ng/mL)	1.3 ± 0.5	0.6 ± 0.3	< 0.01
EAP (ng/mL)	0.7 ± 0.2	0.4 ± 0.1	< 0.01
DHEA (ng/mL)	2.9 ± 1.5	5.1 ± 3.8	< 0.05
DHEAS (μg/mL)	2.4 ± 1.1	2.7 ± 2.0	n.s.

Values are means + S.D. Statistical analysis was performed by Student’s t test

No significant associations were found between blood neurosteroids and BDI, SAS, HIT-6 and MIDAS scores, and educational level in EM patients and controls.

### Chronic migraine (including medication-overuse)

Circulating levels of neurosteroids in CM patients are shown in Table 3. Similarly to EM patients, CM patients showed large increases in AP levels, whereas EAP levels did not change, and levels of *both* DHEA and DHEAS were reduced. A direct comparison of AP, DHEA, and DHEAS levels between patients affected by EM and CM is shown in Table 4. Interestingly, DHEA and DHEAS levels were significantly lower in CM patients with respect to EM patients, whereas AP levels did not differ between the two groups (Table 4). Thus, neurosteroids behaved differently in EM and CM patients, as compared to their respective controls. At least in CM patients, changes in neurosteroid levels were unaffected by the hormonal status, as shown by a subgroup analysis in patients examined before and after menopause, or in fertile patients examined in the follicular and luteal phases of the ovarian cycle (data not shown). Thus, it appears that migraine itself rather than fluctuations in ovarian hormones, is the main driver of neurosteroid changes in CM patients.

**Table 3:** Plasma neurosteroid levels in patients affected by chronic migraine patients and healthy controls

	Chronic migraine (overall population)	Controls	*P*
AP (ng/mL)	1.1 ± 0.3	0.61 ± 0.3	< 0.01
EAP (ng/mL)	0.4 ± 0.2	0.41 ± 01	n.s.
DHEA (ng/mL)	1.6 ± 1.1	5.1 ± 3.8	< 0.01
DHEAS (μg/mL)	1.2 ± 0.9	2.76 ± 2.0	< 0.01

Values are means + S.D. Statistical analysis was performed by Student’s t test

**Table 4:** Comparative analysis of DHEA, DHEAS, and AP levels in patients affected by episodic or chronic migraine

	Episodic migraine (*n* = 19)	Chronic migraine (*n* = 51)	*P*
DHEA (ng/mL)	2.9 ± 1.5	1.6 ± 1.1	< 0.01
DHEAS (μg/mL)	2.4 ± 1.1	1.2 ± 0.9	< 0.01
AP (ng/mL)	1.3 ± 0.5	1.1 ± 0.3	n.s.

Values are means + SD. Statistical analysis was performed by Student’s t test

In the overall population of CM patients neurosteroid levels also did no change when blood samples were collected during a migraine attack or in the interictal period (Table 5). However, changes were found when patients affected by CM + MO were isolated from the overall population of CM patients according to the ICDH3-beta diagnostic criteria. In these patients, AP levels were lower during the headache attack (0.9 ± 0.2 ng/mL vs 1.1 ± 0.2 ng/mL, respectively; *p* < 0.05), whereas in the remaining CM patients, DHEAS levels were higher during the attack (1.8 ± 1.4 μg/mL vs 0.9 ± 0.6 μg/mL, respectively; *p* < 0.05).

**Table 5:** Plasma neurosteroid levels in the overall population of patients affected by chronic migraine during the headache attack and in the interictal period

	Chronic migraine during attack (*n* = 27)	Chronic migraine no attack (*n* = 24)	Controls
AP (ng/mL)	1.1 ± 0.4*	1.1 ± 0.2*	0.6 ± 0.3
EAP (ng/mL)	0.5 ± 0.1	0.4 ± 0.2	0.4 ± 0.1
DHEA (ng/mL)	1.4 ± 0.9*	1.8 ± 1.2*	5.1 ± 3.8
DHEAS (μg/mL)	1.5 ± 1.1*	1.0 ± 0.6*	2.7 ± 2.0

Values are means + S.D. *p<0.05 vs. the respective control groups. No significant changes were found between values obtained during the attack and in the interictal period. Statistical analysis was performed by One-way ANOVA + Fisher’s LSD (AP, F_2,79_ = 24.79; EAP, F_2,79_ = 1.43; DHEA, F_2,79_ = 18.06; DHEAS, F_2,79_ = 10.66)

AP and EAP are known to differentially regulate γ-aminobutyric acid A receptors, with AP activating and EAP inhibiting receptor function, respectively [20]. Hence, we measured the AP/EAP ratio as a surrogate marker of the cumulative effects of these neurosteroids on γ-aminobutyric acid mediated transmission. The AP/EAP ratio was higher In the overall population of CM patients with respect to healty control (3.0 ± 1.7 vs 1.8 ± 1.2, respectively; *p* < 0.01), whether or not blood samples were collected during the headache attack or in the interictal period. The subpopulation of patients with CM + MO showed a trend to a reduction in the AP/EAP ratio during the attack with respect to the interictal period (2.2 ± 1.2 vs 3.4 ± 1.8, respectively; *p* = 0.06).

No significant associations were found between plasma neurosteroid levels and BDI, SAS, HIT-6 or MIDAS scores. In addition, there was no association between neurosteroids and education level in our patients.

### Cluster headache

Patients affected by CH showed lower levels of AP and no changes in other neurosteroids with respect to healthy controls (Table 6). The reduction in AP levels was maintained when blood samples were collected during or between headache attacks (0.4 ± 0.1 ng/mL; *n* = 6, *p* < 0.05 vs controls during attacks, and 0.3 ± 0.1 ng/mL; *n* = 12, vs the same controls, p < 0.05 in the interictal period). The other neurosteroids did not change during or between headache attacks.

**Table 6:** Plasma neurosteroid levels in patients affected by cluster headache and their healthy controls

	Cluster headache	Controls	*P*
AP (ng/mL)	0.3 ± 0.1	0.7 ± 0.2	< 0.01
EAP (ng/mL)	0.4 ± 0.1	0.3 ± 0.2	n.s.
DHEA (ng/mL)	2.9 ± 2.2	4.3 ± 5.0	n.s.
DHEAS (μg/mL)	2.6 ± 1.7	2.2 ± 1.7	n.s.

Values are means + S.D. Statistical analysis was performed by Student’s t test

No significant associations were found between neurosteroids and BDI, SAS or HIT-6 scores or education level also in patients affected by CH.

## Discussion

Our findings suggest that changes in neurosteroid levels contribute to the pathophysiology of migraine and cluster headache, with the obvious limitation that plasma levels may not entirely reflect CNS levels. We found elevated AP levels and reduced DHEA levels in all patients affected by migraine, whereas EAP levels were increased only in patients affected by EM, and DHEAS levels were reduced only in patients affected by CM. It is generally believed that abnormalities in the regulation of neuronal excitability underlie the pathophysiology of migraine, giving raise to cortical spreading depression or to functional changes in pain regulatory centers of the brainstem [21]. Neurosteroids play a key role in the regulation of neuronal excitability by modulating the activity of γ-aminobutyric acid A and N-methyl-d-aspartate receptors [5], with AP behaving as a positive allosteric modulator of both synaptic and extrasynaptic γ-aminobutyric acid A receptors by interacting with a picrotoxin-sensitive site localized within the γ-aminobutyric acid A -gated chloride channel [22, 23]. Activation of γ-aminobutyric acid A receptors restrains synaptic excitation, and, therefore, the increase in AP found in EM and CM patients might be considered as a defensive mechanism aimed at limiting the enhanced neuronal excitability associated with migraine. This putative defensive mechanism might also involve γ-aminobutyric acid A receptors localized outside the blood-brain barrier. Accordingly, Moskowitz et al. have found that systemic injection of progesterone, AP, and other γ-aminobutyric acid-mimetic neurosteroids inhibits neurogenic edema in the rat meninges, and this effect is blocked by bicuculline, a γ-aminobutyric acid A receptor antagonist that is unable to cross the blood-brain barrier [24]. The reduction in DHEA (in EM and CM patients) and DHEAS (only in CM patients) levels might contribute to restrain neuronal excitation and neurogenic edema because both steroids behave as weak negative allosteric modulators or γ-aminobutyric acid A receptors [7]. Interestingly, and counterintuitively, the drop in DHEA and DHEAS levels was more substantial in CM patients, suggesting that adaptive mechanisms that reinforce γ-aminobutyric acid mediated transmission are more prominent in CM with respect to EM. Accordingly, only EM patients showed increases in the levels of EAP, a neurosteroid that is devoid of intrinsic efficacy and behaves as competitive antagonist at the AP site of γ-aminobutyric acid A receptors [20]. Perhaps, it is a high recurrence of migraneous attacks that drives the large reductions in DHEA and DHEAS (associated with the increase in AP) as an extreme but unsuccessful attempt to reinforce γ-aminobutyric acid mediated transmission. In contrast, even a lower reduction in DHEA levels (always associated with the increase in AP levels) might be sufficient to restrain migraneous episodes in patients affected by EM. Interestingly, patients affected by CM + MO showed reduced AP levels and AP/EAP ratio during the migraneous attack, whereas the remaining CM patients showed increases in DHEAS levels during the attack. These findings suggest that either changes in neurosteroid levels are causally related to the onset of the migraneous attack, or, alternatively, is the attack itself that causes alterations in neurosteroid levels. We favour the former hypothesis because a reduction in AP levels (in CM + MO patients) and an increase in DHEAS levels (in non-CM + MO patients) should restrain synaptic inhibition. Longitudinal studies in patients are necessary to establish whether measurements of plasma neurosteroids may help to predict the transition from EM to CM.

A different scenario was observed in patients affected by CH, in which AP levels were largely reduced, and not increased as observed in EM or CM patients. This suggests that changes in neurosteroid levels in CH are not “defensive”, but might contribute to the pathophysiology of CH, lending credit to the hypothesis that migraine and CH are different disease, yet sharing an increased neuronal excitability as a common pathophysiological substrate. The reduction of AP levels could also help to explain the more severe and disabling clinical phenotype of CH, as compared to migraine. Progesterone is a metabolic precursor of both AP and testosterone, and testosterone levels are known to be reduced in CH [9]. No data are available on changes in progesterone levels in CH because the disorder is highly prevalent in males. Perhaps there is a primary reduction in progesterone levels in CH, which results into a secondary drop in both AP and testosterone levels. This interesting hypothesis warrants further investigation.

## Conclusion

In conclusion, we have shown for the first time that large and disease-specific changes in circulating neurosteroid levels are associated with chronic headache disorders, raising the interesting possibility that fluctuations of neurosteroids at their site of action might shape the natural course of migraine and CH. Whether the observed changes in neurosteroids are genetically determined or rather result from exposure to environmental or intrinsic stressors [25] is unknown. This might also be matter for further investigation because stress is a known triggering factor for headache attacks in both migraineurs and CH patients.

In the future, it will be interesting to verify also the correlations between specific comorbidities presenting similar neural hyperexcitability of migraine, like fibromyalgia [26], or correlate the neuroinflammation related to dyslipidemia in migraine [27].

Last, our findings may lay the groundwork for novel neurosteroid-based therapeutic strategies in the treatment chronic headache disorders. Ganaxolone, a synthetic γ-aminobutyric acid-mimetic analogue of AP, has been developed for the treatment of epilepsy, and it showed good efficacy and safety profile in patients with uncontrolled focal seizures in a phase-II placebo controlled clinical trial [28]. Our data encourage the experimental use of ganoxolone particularly in CH patients, where AP levels were found to be largely reduced.

## Abbreviations

AP: Allopregnanolone
BDI Beck: Depression Inventory
CH: Cluster headache
CM: Chronic migraine
CNS: Central Nervous System
CONSORT: Consolidated Standards of Reporting Trials
DHEA: Dehydroepiandrosterone
DHEAS: Dehydroepiandrosterone sulfate
EAP: Epiallopregnanolone
EM: Episodic migraine
HIT-6: Headache impact test
ICHD3 ß: International Classification of Headache Disorders 3rd Edition ß version
LC-MS/MS: Liquid chromatography tandem mass spectrometry
MIDAS: Migraine disability assessment test
MOH: Medication overuse headache
SAS: Self-rating anxiety scale
